# Antibacterial Activity of Glutathione-Stabilized Silver Nanoparticles Against *Campylobacter* Multidrug-Resistant Strains

**DOI:** 10.3389/fmicb.2018.00458

**Published:** 2018-03-16

**Authors:** Jose M. Silvan, Irene Zorraquin-Peña, Dolores Gonzalez de Llano, M. Victoria Moreno-Arribas, Adolfo J. Martinez-Rodriguez

**Affiliations:** ^1^Grupo de Microbiología y Biocatálisis de Alimentos, Departamento de Biotecnología y Microbiología de Alimentos, Instituto de Investigación en Ciencias de la Alimentación, CSIC-UAM, Madrid, Spain; ^2^Grupo de Biotecnología Enológica Aplicada, Departamento de Biotecnología y Microbiología de Alimentos, Instituto de Investigación en Ciencias de la Alimentación, CSIC-UAM, Madrid, Spain

**Keywords:** *Campylobacter*, silver nanoparticles, antibacterial activity, antibiotic resistance, multidrug resistant strains

## Abstract

*Campylobacter* is the leading cause of bacterial diarrheal disease worldwide. Although most episodes of campylobacteriosis are self-limiting, antibiotic treatment is usually needed in patients with serious enteritis, and especially in childrens or the elderly. In the last years, antibiotic resistance in *Campylobacter* has become a major public health concern and a great interest exists in developing new antimicrobial strategies for reducing the impact of this food-borne pathogen on human health. Among them, the use of silver nanoparticles as antibacterial agents has taken on increased importance in the field of medicine. The aim of the present study was to evaluate the antimicrobial effectiveness of glutathione-stabilized silver nanoparticles (GSH-Ag NPs) against multidrug resistant (MDR) *Campylobacter* strains isolated from the chicken food chain (FC) and clinical patients (C). The results obtained showed that GSH-Ag NPs were highly effective against all MDR *Campylobacter* strains tested. The minimal inhibitory concentration (MIC) and minimal bactericidal concentration (MBC) were in a range from 4.92 to 39.4 μg/mL and 9.85 to 39.4 μg/mL, respectively. Cytotoxicity assays were also assessed using human intestinal HT-29, Caco-2, and CCD-18 epithelial cells. Exposure of GSH-Ag NPs to intestinal cells showed a dose-dependent cytotoxic effect in all cell lines between 9.85 and 39.4 μg/mL. More than 60% of the tested *Campylobacter* strains were susceptible to GSH-Ag NPs concentrations ≤ 9.85 μg/mL, suggesting that practical inhibitory levels could be reached at low GSH-Ag NPs concentrations. Further works are needed with the purpose to evaluate the practical implications of the toxicity studies and to know more about other attributes linked to the biological compatibility. This behavior makes GSH-Ag NPs as a promising tool for the design of novel antibacterial agents for controlling *Campylobacter*.

## Introduction

*Campylobacter* is the leading cause of bacterial food-borne gastroenteritis worldwide and more than 95% of the infections attributed to this genus are associated with the species *Campylobacter jejuni* (*C. jejuni*) and *Campylobacter coli* (*C. coli*) (Ganan et al., 2012). Campylobacteriosis has been the most frequently reported cause of human food-borne zoonoses in the EU since 2004 (European Food Safety Authority European Centre for Disease Prevention Control, 2016). Patients may experience mild to severe illness, and symptoms can include gastrointestinal manifestations such as diarrhea, abdominal cramps, nausea, and fever. The severity of symptoms during the disease mainly depends on the infective strain and on the medical condition of the patient (Blaser and Engberg, 2008). Bacteraemia and other extra intestinal complications may develop less frequently. In a reduced percentage of cases, potentially severe long-term complications may occur, such as Guillain-Barré syndrome, Reiter's syndrome or reactive arthritis (Kaakoush, 2015; O'Brien, 2017). Although most cases of campylobacteriosis are self-limiting, antibiotic therapy is generally used in cases with severe or long-lasting enteritis, especially in children and the elderly, immunocompromised patients, and in cases of extra intestinal manifestations. However, antimicrobial resistance in bacteria from food of animal origin, including *Campylobacter*, has become in the last years a serious public health concern in both developed and developing nations. A rising amount of *Campylobacter* isolates have become resistant to different antibiotic families such as fluoroquinolones, aminoglycosides, macrolides, and beta-lactams among others (Wieczorek and Osek, 2013; Bolinger and Kathariou, 2017; European Food Safety Authority European Centre for Disease Prevention Control, 2017). The increase in the incidence of infections caused by multidrug resistant (MDR) strains [lower susceptibility to at least three antibiotic families according to epidemiological cut-off values (ECOFFs)] of *Campylobacter* makes the treatment of this disease increasingly complicated (European Food Safety Authority European Centre for Disease Prevention Control, 2017). For these reasons, it is necessary to find new alternatives to the use of antibiotics in the control of *Campylobacter*.

Improvement of conventional antimicrobials by new technologies to transcend antimicrobial resistance is in development. Nanotechnology-driven innovations offer new perspectives for both patients and professionals to tackle drug resistance. Previous works have shown that antimicrobial formulations in the nanoparticles format could be employed as effective bactericidal materials due to their enhanced reactivity, resulting from their high surface/volume ratio (Choi et al., 2008; Rudramurthy et al., 2016). Particularly, silver nanoparticles are reported to exhibit strong biocidal properties on different bacterial species (Quelemes et al., 2013; Losasso et al., 2014), including MDR bacteria (Lara et al., 2010). In recent years, the use of silver nanoparticles as antibacterial agents has become more important in the medical field (Marambio-Jones and Hoek, 2010), due to the importance to provide alternatives to the resistance that many pathogenic microorganisms exhibit to most widely used antibiotics. Silver nanoparticles can be coated to facilitate their interaction with the environment. In this sense, a coating of glutathione (GSH) increases the solubility and the ability of silver nanoparticles to interact with the environment. However, there are no previous studies about the impact of silver nanoparticles on *Campylobacter*, in spite of its importance as a food-borne pathogen. On the other hand, cytotoxicity of nanoparticles is a major concern in the use and development of nanotechnology. Toxicity of nanoparticles to eukaryotic cells is associated to the higher reactivity of these particles due to their large surface (Fröhlich and Fröhlich, 2016). In general, *in vitro* studies suggest that silver nanoparticles may induce cellular death, increased reactive oxygen species (ROS) production, oxidative stress, and DNA damage (Kim and Ryu, 2013). Despite, the scientific evidence on potential adverse effects of nanoparticles severely lags behind the advances of nanotechnologies. For these reasons, the main objective of the present work was to evaluate the *in vitro* antimicrobial activity of GSH-Ag NPs against several strains of *Campylobacter*, also studying the effects of these nanoparticles on different human intestinal cell lines.

## Materials and methods

### Materials and reagents

3,4,5-dimethylthiazol-2,5-diphenyl-tetrazolium bromide (MTT), dimethyl sulfoxide (DMSO), reduced glutathione, silver perchlorate, and silver tetrafluoroborate were acquired from Sigma-Aldrich (Madrid, Spain). Dulbecco's Modified Eagle's Medium (DMEM), penicillin/streptomycin (5,000 U/mL), phosphate buffered saline (PBS) and trypsin/EDTA solution (170,000 U/L) were purchased from Lonza (Barcelona, Spain). Fetal bovine serum (FBS) of South American origin (Hyclone, South Logan, UT, USA) was obtained from Thermo Scientific (Waltham, MA USA). Cell culture dishes were obtained from Sarstedt (Nümbrecht, Germany).

### Synthesis of glutathione-stabilized silver nanoparticles (GSH-Ag NPs)

**Figure 1:**
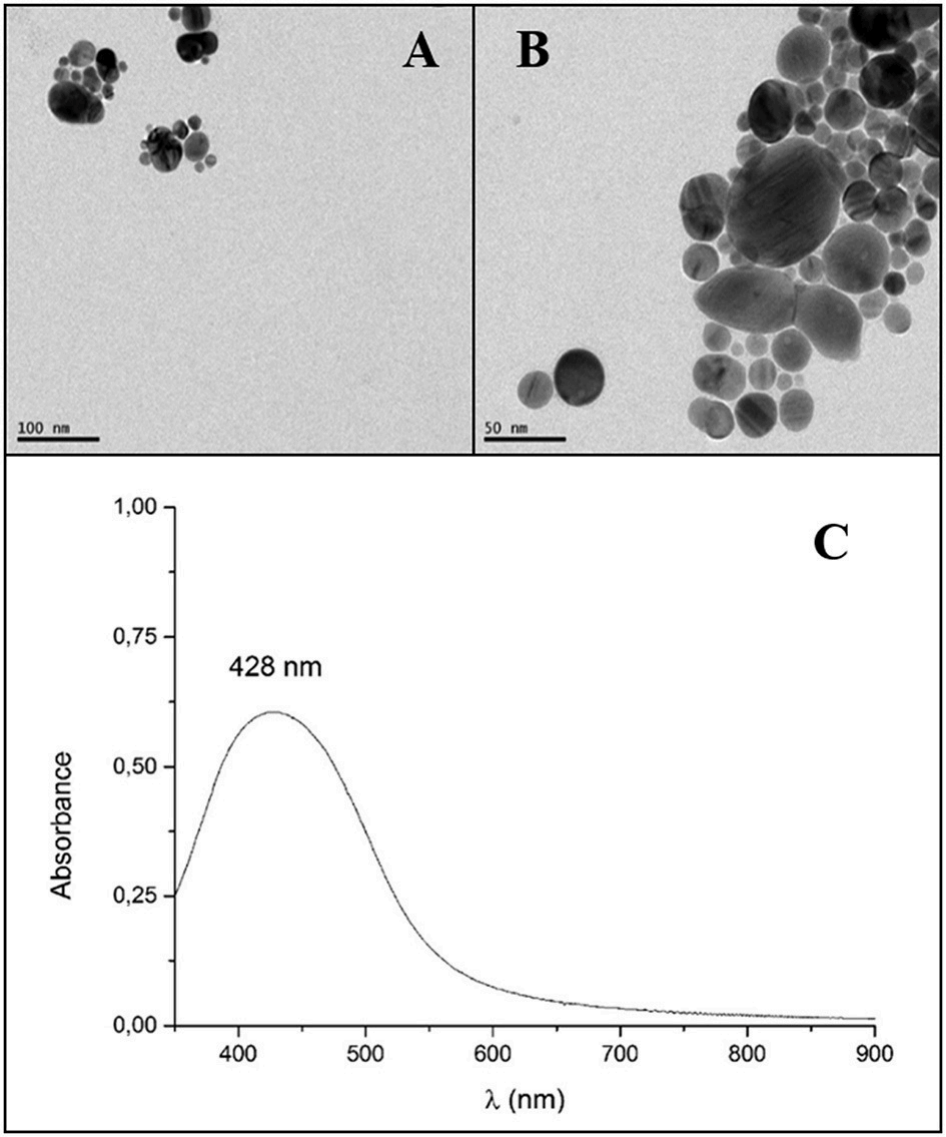
Microscopy characterizations of glutathione-stabilized silver nanoparticles (GSH-Ag NPs). Transmission electron microscopy images (TEM) of GSH-Ag NPs. Scale bars are 100 nm **(A)** and 50 nm **(B)**. UV-Vis absorption spectra of GSH-Ag NPs in water **(C)**.

GSH-Ag NPs used in this work were synthetized following a classical approach as described in García-Ruiz et al. (2015). Briefly, AgBF4 (0.096 mmol, 18.7 mg) was dissolved in 50 mL of water. This silver salt was reduced using NaBH4 (0.026 mmol, 0.001 g) with vigorous stirring at room temperature. After 30 min of stirring, 2 mL of a 10^−2^ M glutathione solution in water was added dropwise to the silver colloidal solution. The formed glutathione-stabilized silver nanoparticles solution was kept in the dark. The obtained nanoparticles showed a heterogeneous range of diameters between 10 and 50 nm, and a final concentration of silver of 0.197 mg/mL. The UV-Vis spectrum of water solutions of GSH-Ag NPs displayed an intense and sharp localized surface plasmon resonance (LSPR) band at 429 nm (Figure 1). Both characteristics of the absorption band associated with surface plasmon resonance are due to the GSH complex in accordance with the size and dispersion of sizes obtained with the nanoparticles stabilized with glutathione (García-Ruiz et al., 2015).

### Bacterial strains, growth media, and culture conditions

The Burgos University kindly donated the different *Campylobacter* strains used in this work. These strains were isolated from different sections of the chicken meat food chain and from cases diagnosed with campylobacteriosis at Burgos University Hospital. *C. jejuni* 11168 obtained from National Collection of Type Cultures (NCTC) (London, UK) was used as reference strain. The isolation source, species, strain designation and isolation place of strains used in this study are shown in Table 1. All isolates were performed in the province of Burgos (Spain) between the years 2011 and 2014, and stored at −80°C in Brucella Broth (BB) (Becton- Dickinson, NJ, USA) plus 20% of glycerol until were used. The agar medium consisted of Müeller-Hinton agar supplemented with 5% defibrinated sheep blood (MHB) (Becton-Dickinson). Liquid growth medium for *Campylobacter* strains consisted of BB. The frozen strains were propagated by inoculation in MHB and incubation under microaerophilic conditions (85% N_2_, 10% CO_2_, and 5% O_2_) using a Variable Atmosphere Incubator (VAIN) (MACS-VA500, Don Whitley Scientific, Shipley, UK) at 40°C for 48 h. Isolated colonies were inoculated into 50 mL of BB and incubated under stirring at 150 rpm on an orbital shaker at 40°C for 24 h in microaerophilic conditions in the VAIN. These bacterial inoculum cultures (~1 × 10^8^ colony forming units (CFU)/mL) were used for the different experimental assays. BSL2 facilities of CIAL were used for the development of the proposed work.

**Table 1:** *Campylobacter* strains used in the present study.

**Isolation source**	**Strain designation**	**Specie**	**Isolation place**	**Isolation source**	**Strain designation**	**Specie**	**Isolation place**
Chicken Food Chain (FC)	FC1	*C. jejuni*	Cloacal swab slaughterhouse	Clinical (C)	C1	*C. jejuni*	Pediatric unit
	FC2	*C. jejuni*	Defeathering machine		C2	*C. jejuni*	Pediatric unit
	FC3	*C. jejuni*	Dirty crate		C3	*C. jejuni*	Pediatric unit
	FC4	*C. jejuni*	Clean crate		C4	*C. jejuni*	Digestive unit
	FC5	*C. coli*	Hamburger retail		C5	*C. coli*	Pediatric unit
	FC6	*C. coli*	Drumstick retail		C6	*C. coli*	Internal medicine unit
	FC7	*C. coli*	Cutting table		C7	*C. coli*	Internal medicine unit
	FC8	*C. coli*	Clean mince machine		C8	*C. coli*	Digestive unit
	FC9	*C. jejuni*	Boot sock		C9	*C. coli*	Pediatric unit
	FC10	*C. jejuni*	Fecal swab		C10	*C. jejuni*	Pediatric unit
	FC11	*C. jejuni*	Fecal swab		C11	*C. jejuni*	Pediatric unit
	FC12	*C. jejuni*	Cutting table		C12	*C. jejuni*	Pediatric unit
	FC13	*C. jejuni*	Breast deboning		C13	*C. jejuni*	Pediatric unit
	FC14	*C. coli*	Carcass before chilling		C14	*C. jejuni*	Pediatric unit
	FC15	*C. jejuni*	Drumstick retail		C15	*C. jejuni*	Pediatric unit
	FC16	*C. coli*	Hamburger retail		C16	*C. coli*	Surgery unit
	FC17	*C. coli*	Hamburger retail		C17	*C. coli*	Pediatric unit
	FC18	*C. coli*	Hamburger retail		C18	*C. coli*	Microbiology unit
	FC19	*C. jejuni*	Clean mince machine		C19	*C. coli*	Traumatology unit
	FC20	*C. jejuni*	Hamburger retail		C20	*C. coli*	Gastroenterology unit

*All the strains used are from the CIAL collection of microbial cultures*.

### Antibiotic susceptibility test

The antibiotic susceptibility was assessed following the Kirby-Bauer disc diffusion method based on the performance standards for antimicrobial disk susceptibility test described by Clinical and Laboratory Standards Institute (Clinical Laboratory Standards Institute, 2012). Antimicrobial discs (Oxoid, Basingstoke, UK) were placed on the inoculated MHB plates and they were incubated in the VAIN for 48 h. Nine antibiotics from the most frequently used against *Campylobacter*, representing five different families, were used: macrolides (erythromycin, 15 μg), quinolones (nalidixic acid 30 μg) and fluoroquinolones (norfloxacin 10 μg; ciprofloxacin 5 μg), tetracyclines (tetracycline; 30 μg), aminoglycosides (streptomycin 25 μg; gentamicin 10 μg), and β-lactam antibiotics (ampicillin 10 μg; amoxicillin-clavulanic acid 30 μg). The control strain used was *C. jejuni* NCTC 11351. Media, incubation times and temperature used for campylobacters were the same described above. Breakpoints used were chosen based on the antibiotic tested. Interpretation of the results for ciprofloxacin, erythromycin, and tetracycline was performed using the resistance breakpoint for campylobacters according to The European Committee on Antimicrobial Susceptibility Testing (2018). Breakpoint for amoxicillin-clavulanic acid was evaluated in accordance with interpretive criteria provided by the Comite' de l'antibiogramme de la Societe' Francaise de Microbiologie (2017). Breakpoints used for nalidixic acid, norfloxacin, gentamicin and ampicillin were those reported by Luangtongkum et al. (2007). In the case of streptomycin, no breakpoints were available for campylobacters, and susceptibility categorization was carried out using the breakpoints established by CLSI for the family *Enterobacteriaceae*, as reported by others (Giacomelli et al., 2014).

### Antibacterial activity of GSH-Ag NPs against *Campylobacter* strains

The antibacterial activity of GSH-Ag NPs against *C. jejuni* 11168 was evaluated following the procedure described by Silvan et al. (2013). Briefly, 1 mL GSH-Ag NPs (0.61–39.4 μg/mL final concentrations) was transferred into different flasks containing 4 mL of BB. Bacterial inoculum (50 μL of ~1 × 10^8^ CFU/mL) was inoculated into the flasks under aseptic conditions. Culture was prepared in triplicate and incubated microaerobically under stirring at 150 rpm at 40°C for 24 h in the VAIN. Positive growth controls (bacteria without nanoparticles) were prepared by transferring 1 mL of sterile water to 4 mL of BB and 50 μL of bacterial inoculum. After incubation, serial decimal dilutions of mixtures were prepared in saline solution (0.9% NaCl) and they were plated (20 μL) onto fresh MHB agar and incubated microaerobically at 40°C in the VAIN. The CFU was assessed after 48 h of incubation. Results were expressed as log CFU/mL.

A micromethod was used for the assay with the different *Campylobacter* strains. With this purpose, the minimal inhibitory concentration (MIC) and the minimal bactericidal concentration (MBC) were determined as follow: BB (240 μL), GSH-Ag NPs (60 μL) at different concentrations and bacterial inoculum (3 μL of ~1 × 10^8^ CFU/mL) were dispensed into sterile 96-well flat-bottom microplate. A control growth (bacteria without nanoparticles) for each strain was also prepared. Microplate was incubated microaerobically under stirring at 150 rpm at 40°C for 24 h in the VAIN. In order to determine the MIC and MBC, 5 μL of culture from each well were plated onto MHB and incubated microaerobically at 40°C for 48 h in the VAIN. MIC was defined as the lowest amount of GSH-Ag NPs that provokes a decrease in viability respect to the control growth (visual reduction of growth) after 24 h of treatment (calculated in approximately 4 log of inhibition). MBC was defined as the lowest bactericidal concentration of GSH-Ag NPs after 24 h of treatment. Results were expressed as μg/mL.

### Cytotoxicity of GSH-Ag NPs

The cell viability was determined by the MTT reduction assay in colon tumoral cell lines (HT-29 and Caco-2) and colon regular cell line (CCD-18) obtained from American Type Culture Collection (ATCC) (Manassas, VA, USA). Cells were cultured in DMEM supplemented with 10% FBS and 1% penicillin/streptomycin. Cells were plated at densities 1 × 10^5^ cells in 75 cm^2^ tissue culture flasks and maintained at 37°C under 5% CO_2_ in a humidifier atmosphere. The culture medium was changed every two days. Confluent stock cultures were trypsinized (Trypsin/EDTA) and cells were seeded in 96-well plates (~5 × 10^4^ cells per well) and incubated in culture medium at 37°C under 5% CO_2_ in a humidifier incubator for 24 h.

Briefly, cell medium was replaced with serum-free medium containing different concentrations of GSH-Ag NPs (0.61–39.4 μg/mL final concentrations) and the cells were incubated at 37°C under 5% CO_2_ for 24 h. Control cells were incubated in serum-free medium without GSH-Ag NPs addition. The cells were then washed twice with PBS and added 200 μL of serum-free medium. Thereafter, 20 μL of a MTT solution in PBS (5 mg/mL) was added to each well for the quantification of the living metabolically active cells after 1 h of incubation. MTT is reduced to purple formazan in the mitochondria of living cells. Culture medium was removed and formazan crystals formed in the wells were solubilized in 200 μL of DMSO. Absorbance was measured at 570 nm wavelength employing a microplate reader Synergy HT (BioTek Instruments, Winooski, VT, USA). The viability was calculated considering control cells incubated with serum-free medium as 100% viable. Data represent the mean and standard deviation of three independent experiments (*n* = 3). All experiments were carried out between passages 10–30 to ensure cell uniformity and reproducibility.

### Statistical analysis

The results were reported as means ± standard deviations (SD) performed in triplicate. The data were subjected to statistical analysis by one-way analysis of variance (ANOVA) followed by Dunnett's method for multiple comparisons. Differences were considered significant at *p* < 0.05. All statistical tests were performed with IBM SPSS Statistics for Windows, Version 21.0 (IBM Corp., Armonk, NY, USA).

## Results and discussion

### Antibacterial activity of GSH-Ag NPs against *C. jejuni* 11168

**Figure 2:**
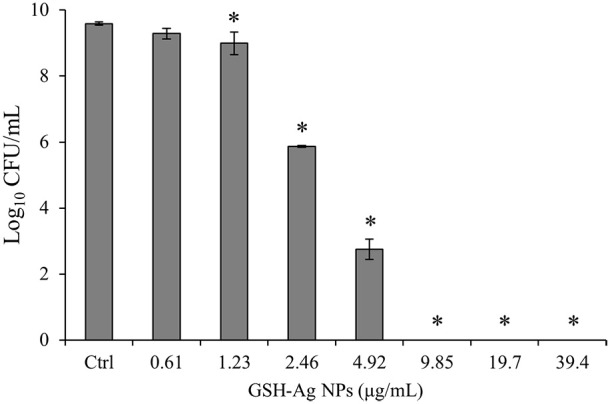
Antibacterial activity of glutathione-stabilized silver nanoparticles (GSH-Ag NPs) against *C. jejuni* 11168. Results represent the mean ± SD of Log_10_ CFU/mL (*n* = 3). Bars marked with asterisk indicate significant differences (*p* < 0.05) compared to the control growth (sample without nanoparticles) by one-way analysis of variance (ANOVA), followed by Dunnett's method for multiple comparisons.

The results of the antibacterial properties of GSH-Ag NPs against *C. jejuni* 11168 are showed in the Figure 2. The nanoparticles, in a final concentration range of 9.85–39.4 μg/mL, were bactericidal after 24 h of incubation. Small concentrations of GSH-Ag NPs (1.23 and 4.92 μg/mL) significantly (*p* < 0.05) inhibited the growth of *C. jejuni* 11168 strain. These results demonstrate the strong capacity of the GSH-Ag NPs with a range of 10–50 nm of particle size to inhibit *Campylobacter* growth. Previous studies have described a greater susceptibility of some food-borne pathogens such as *Escherichia coli, Listeria monocytogenes, Pseudomonas aeruginosa*, or *Salmonella* to silver nanoparticles (Crespo et al., 2012; Taglietti et al., 2012; Tamboli and Lee, 2013). Ag^+^ released from nanoparticles reacts with sulfur-containing proteins, mainly on the cell surface, and phosphorous-containing nucleic acids. They are known to produce ROS inside the cell, eventually leading to cell death (Rudramurthy et al., 2016). It is well known that the differences in the material employed in the synthesis of the nanoparticles can play an important role in their antimicrobial activity. The biological application for silver nanoparticles requires an appropriate coating of nanoparticle surface, because it could favor interactions with biosystems and enhance the solubility in water-based environments. GSH has proved to be a good candidate for this purpose since this biomolecule displays a thiolic function, capable of being anchored to silver surfaces, and the presence of functional groups (carboxylates and amine) that promote water solubility and interactions toward more complex biostructures (Taglietti et al., 2012). These GSH-Ag NPs have proved to be more effective for Gram negative bacteria, possibly because their cell wall contains a thinner peptidoglycan layer than Gram positive bacteria (Taglietti et al., 2012; García-Ruiz et al., 2015). Also, it is well known that the particle size and distribution can play an important role in their antimicrobial activity. It has been described that nanoparticles with a smaller size tend to be more effective as antimicrobials (Gogoi et al., 2006). However, in this work the antibacterial effect of GSH-Ag NPs against *C. jejuni* 11168 was higher than those reported in previous studies against other bacteria using smaller sized silver nanoparticles (Guzman et al., 2012; Losasso et al., 2014).

### Antibiotic susceptibility test of *Campylobacter* strains

The results of the antimicrobial resistance of *Campylobacter* strains are presented in Table 2. All isolates were susceptible to erythromycin, except the clinical strains C5 and C19 (*C. coli*). Erythromycin is the first therapeutic option for the treatment of severe *Campylobacter* infections, thus the prevalence of resistance to this antimicrobial drug should be a cause for particular concern. Studies on the susceptibility of *Campylobacter* strains to macrolides, such as erythromycin, have been shown that the percentage of resistant strains is currently at a low level (Wieczorek and Osek, 2015; Bolinger and Kathariou, 2017). However, recent reports have documented the emergence of some *Campylobacter* strains showing erythromycin resistance (Florez-Cuadrado et al., 2016; Bolinger and Kathariou, 2017). In some European countries, up to a third to half of *C. coli* isolated from humans were resistant to erythromycin (European Food Safety Authority European Centre for Disease Prevention Control, 2017). In this work, two clinical isolates of *C. coli* strain were resistant to erythromycin, suggesting that *C. coli* may represent an underestimated potential health risk for consumers. Most of the strains (97.5%) were resistant to nalidixic acid, norfloxacin, ciprofloxacin and tetracycline (Table 2). It has been previously defined that resistance to these antimicrobials is predominant among strains of *Campylobacter* from poultry meat, finding also in clinical cases very high proportions of strains resistant to ciprofloxacin and tetracyclines (European Food Safety Authority European Centre for Disease Prevention Control, 2017). Considered in the past as one of the most effective antibiotics against *Campylobacter*, nowadays the level of acquired resistance to fluoroquinolones preclude the use of these antimicrobial agents for routine empirical treatment of human campylobacteriosis (European Food Safety Authority European Centre for Disease Prevention Control, 2017). As expected, a high level of resistance was found for ampicillin (67.5%) and somewhat more moderate resistance level was found for amoxicillin/clavulanic acid (32.5%). However, resistance levels found for streptomycin (35%) were relatively high compared to those commonly reported (European Food Safety Authority European Centre for Disease Prevention Control, 2017), and all resistant strains, both from the chicken meat food chain and hospital isolates, were *C. coli*. This may be due to the clonal expansion of resistant populations, and is in agreement with growing concern about the emergence of *C. coli* strains with high rates of antibiotic resistance (European Food Safety Authority European Centre for Disease Prevention Control, 2017). Only one strain (*C. coli* C5) was resistant to gentamicin, which is consequent with the low resistance levels described for this antibiotic. All *Campylobacter* isolates tested showed resistance to three or more antimicrobials families used in the study. These strains can be considered as multidrug resistant (MDR), defined as those strains with resistance or non-susceptibility to at least three different antimicrobial classes (Magiorakos et al., 2012). In all these strains, most of which possessed multidrug resistance, were evaluated the antimicrobial effect of GSH-Ag NPs.

**Table 2:** Antibiotic resistance profile of *Campylobacter* strains isolated from chicken food chain (FC) and campylobacteriosis patients (C).

**Bacterial strains**	**Ery**	**Nal**	**Tetr**	**Nor**	**Cip**	**Amp**	**AmoxiClav**	**Strep**	**Gent**	**Antibiotic resistance**
FC1	−	+	+	+	+	+	−	−	−	(5/9)
FC2	−	+	−	+	+	+	+	−	−	(5/9)
FC3	−	+	+	+	+	+	−	−	−	(5/9)
FC4	−	+	+	+	+	+	−	−	−	(5/9)
FC5	−	+	+	+	+	−	−	+	−	(5/9)
FC6	−	+	+	+	+	−	−	+	−	(5/9)
FC7	−	+	+	+	+	−	−	+	−	(5/9)
FC8	−	+	+	+	+	−	−	+	−	(5/9)
FC9	−	+	+	+	+	+	−	−	−	(5/9)
FC10	−	+	+	+	+	+	+	−	−	(6/9)
FC11	−	+	+	+	+	+	−	−	−	(5/9)
FC12	−	+	+	+	+	+	−	−	−	(5/9)
FC13	−	+	+	+	+	+	−	+	−	(6/9)
FC14	−	+	+	+	+	−	−	+	−	(5/9)
FC15	−	+	+	+	+	−	+	−	−	(5/9)
FC16	−	+	+	+	+	−	−	+	−	(5/9)
FC17	−	+	+	+	+	−	−	+	−	(5/9)
FC18	−	+	+	+	+	−	−	+	−	(5/9)
FC19	−	+	+	+	+	+	+	−	−	(6/9)
FC20	−	+	+	+	+	+	−	−	−	(5/9)
C1	−	+	+	+	+	+	+	−	−	(6/9)
C2	−	+	+	+	+	+	+	−	−	(6/9)
C3	−	+	+	+	+	+	+	−	−	(6/9)
C4	−	+	+	+	+	+	−	−	−	(5/9)
C5	+	+	+	+	+	+	−	−	+	(7/9)
C6	−	−	+	−	−	+	−	+	−	(3/9)
C7	−	+	+	+	+	−	−	+	−	(5/9)
C8	−	+	+	+	+	+	−	+	−	(6/9)
C9	−	+	+	+	+	+	+	−	−	(6/9)
C10	−	+	+	+	+	+	−	−	−	(5/9)
C11	−	+	+	+	+	+	−	−	−	(5/9)
C12	−	+	+	+	+	+	−	−	−	(5/9)
C13	−	+	+	+	+	+	+	−	−	(6/9)
C14	−	+	+	+	+	+	−	−	−	(5/9)
C15	−	+	+	+	+	−	+	−	−	(5/9)
C16	−	+	+	+	+	−	−	+	−	(5/9)
C17	−	+	+	+	+	−	−	−	−	(4/9)
C18	−	+	+	+	+	+	+	−	−	(6/9)
C19	+	+	+	+	+	+	+	−	−	(7/9)
C20	−	+	+	+	+	+	+	+	−	(7/9)
Resistant	2/40	39/40	39/40	39/40	39/40	27/40	13/40	14/40	1/40	
strains	(5.0%)	(97.5%)	(97.5%)	(97.5%)	(97.5%)	(67.5%)	(32.5%)	(35.0%)	(2.5%)	

*(−) Sensitive to antibiotic treatment; (+) Resistance to antibiotic treatment. Ery, Erythromycin; Nal, Nalidixic acid; Tetr, Tetracyclin; Nor, Norfloxacin; Cip, Ciprofloxacin; Amp, Ampicillin; AmoxiClav, Amoxicillin + Clavulanic acid; Strep, Streptomycin; Gent, Gentamicin*.

### Antibacterial activity of GSH-Ag NPs against food chain and clinical *Campylobacter* strains

Table 3 shows the antibacterial activity of GSH-Ag NPs against *Campylobacter* MDR strains of different origins and species. The antibacterial effect of GSH-Ag NPs had a strain-dependent character. GSH-Ag NPs were bactericidal for most of the strains (87.5%) in a MBC range from 19.7 to 39.4 μg/mL. Food chain isolates showed a higher susceptibility to GSH-Ag NPs (60% isolates with MBC between 9.85 and 19.7 μg/mL) than clinical isolates (100% isolates with MBC between 19.7 and 39.4 μg/mL), suggesting that clinical isolates can be better adapted to counteract the GSH-Ag NPs effect. We have seen in these clinical strains a higher resistance to hydrogen peroxide and oxidative stress than in food chain isolates (unpublished data). Although the antibacterial mechanisms of nanoparticles are still unclear, at least four fundamental pathways in the mechanism of action of silver nanoparticles have been considered: they can adhere to microbial cell surface, resulting in membrane damage and changes in transport activity. They can penetrate inside the cell, affecting the cellular machinery. In addition, they can modulate cellular signal system causing cell death, and finally, they can cause increase in reactive oxygen species (ROS) inside the microbial cells leading to cell damage (Dakal et al., 2016). This last point is consequent with the results obtained in the present work and with many studies that attribute the antibacterial activity of silver nanoparticles to oxidative stress or ROS, including hydrogen peroxide (Wang et al., 2017). No differences were found among species, noting that *C. jejuni* and *C. coli* strains showed a similar MBC range (from 9.85 to 39.4 μg/mL), and the slight variation observed was mainly due to the strain tested.

**Table 3:** Antibacterial activity of GSH-Ag NPs against *Campylobacter* strains determined using microtiter drop plate method.

**Food chain strains**	**MIC (μg/mL)**	**MBC (μg/mL)**	**Clinical strains**	**MIC (μg/mL)**	**MBC (μg/mL)**
FC1	19.7	19.7	C1	9.85	19.7
FC2	9.85	9.85	C2	19.7	39.4
FC3	9.85	9.85	C3	9.85	39.4
FC4	9.85	9.85	C4	19.7	19.7
FC5	39.4	39.4	C5	19.7	19.7
FC6	4.92	9.85	C6	9.85	19.7
FC7	4.92	9.85	C7	9.85	19.7
FC8	9.85	19.7	C8	19.7	19.7
FC9	19.7	19.7	C9	19.7	19.7
FC10	19.7	19.7	C10	19.7	19.7
FC11	39.4	39.4	C11	19.7	39.4
FC12	19.7	39.4	C12	19.7	19.7
FC13	19.7	39.4	C13	19.7	19.7
FC14	19.7	39.4	C14	19.7	39.4
FC15	19.7	39.4	C15	19.7	19.7
FC16	19.7	19.7	C16	19.7	39.4
FC17	19.7	39.4	C17	39.4	39.4
FC18	19.7	19.7	C18	39.4	39.4
FC19	9.85	19.7	C19	19.7	39.4
FC20	19.7	39.4	C20	39.4	39.4

***MIC**_50_ = 19.7 μg/mL; **MIC**_90_ = 39.4 μg/mL. MIC, minimal inhibitory concentration; MBC, minimal bactericidal concentration; **MIC**_50_,MIC at which 50% of the isolates are inhibited; **MIC**_90_, MIC at which 90% of the isolates are inhibited; FC, food chain strains; C, clinical strains*.

The MICs showed a similar behavior than MBC and were strain-dependent. Most of the strains (82.5%) had a MIC range from 9.85 to 19.7 μg/mL, being the MIC50 (MIC at which 50% of the isolates are inhibited respect to control growth) of 19.7 μg/mL and the MIC90 (MIC at which 90% of the isolates are inhibited respect to control growth) of 39.4 μg/mL. As same as MBC, the clinical isolates showed a higher MIC (80% isolates with MCI range of 19.7–39.4 μg/mL) than most of the food chain isolates (80% isolates with MCI range of 9.85–19.87 μg/mL).

The current search for new and effective bactericidal compounds is a significant goal with the purpose to fight against MDR strains, and nanoparticles have been established to date as a promising approach to deal with this problem. Silver nanoparticles have shown to be effective against others MDR bacteria, such as *P. aeruginosa*, methicillin resistant *S. aureus* (MRSA)*, A. baumanni*, and *K. pneumoniae* (Leid et al., 2012; Kasithevar et al., 2017). Although both, chromosomal and plasmid-mediated silver resistance are known in bacteria, the fact that silver nanoparticles likely possesses several bactericidal mechanisms in parallel, may explain why bacterial resistance to silver nanoparticles is rare (Natan and Banin, 2017), making its use a promising alternative to cope with MDR strains. This is especially interesting for campylobacteriosis treatment, taking in account that the antibiotics currently used are becoming less effective in the last years.

### Effect of GSH-Ag NPs in the viability of human intestinal cells

**Figure 3:**
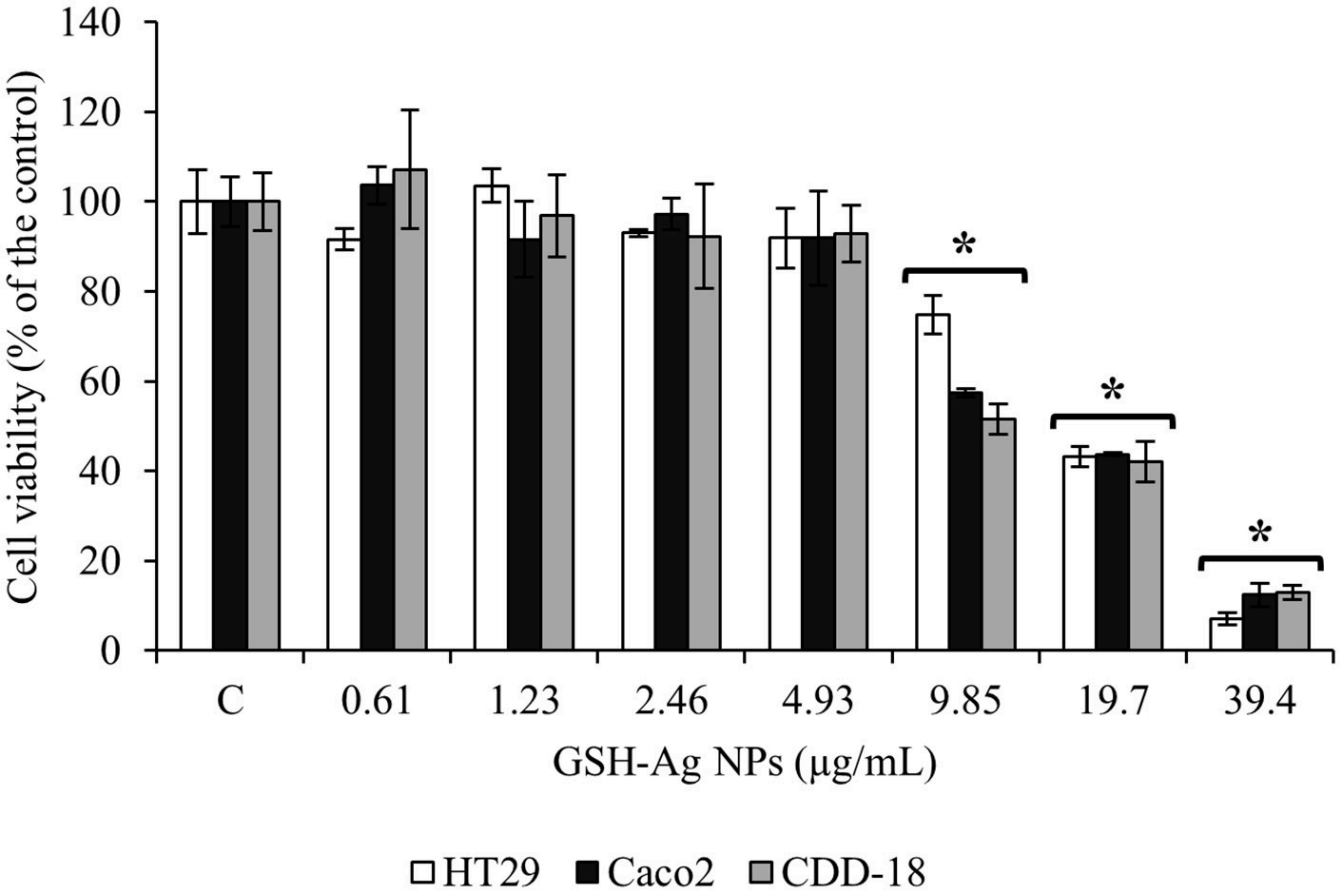
Cytotoxic effects of glutathione-stabilized silver nanoparticles (GSH-Ag NPs) on HT-29, Caco-2, and CCD-18 human intestinal cells. Cells were treated for 24 h, and cell viability was assessed by MTT assay. The results are expressed as percentage of control (cells without nanoparticles) and are represented by mean ± SD (*n* = 3). Bars marked with asterisk indicate significant differences (*p* < 0.05) compared to the control group by one-way analysis of variance (ANOVA), followed by Dunnett's method for multiple comparisons.

*In vitro* experiments using human intestinal epithelial cells facilitate initial investigations into the toxicity of exposures and results inform *in vivo* experiments. This is especially important in the case of nanoparticles ingestion, which is a poorly understood route of exposure. In this work, human intestinal cell lines HT-29, Caco-2, and CCD-18 were used and seven different concentrations of the GSH-Ag NPs (from 0.61 to 39.4 μg/mL) were assayed. Similar viability was observed in all cells lines for the different GSH-Ag NPs concentrations tested. Exposure of GSH-Ag NPs to epithelial cells showed a dose-dependent cytotoxic effect (Figure 3). GSH-Ag NPs concentration up to 4.93 μg/mL showed no significant toxicity (*p* > 0.05). However, cell viability impairment was observed at GSH-Ag NPs concentrations greater than 9.85 μg/mL, reaching the intestinal epithelial cells a death rate ≥ 30% (Figure 3). This behavior is in accordance with most of the reports for silver nanoparticles toxicity, which use to be in the range of 10–100 μg/mL (Chernousova and Epple, 2013). Cytotoxicity is one of the major concerns in the development of silver nanoparticles, sometimes with controversial results, because many studies consist of a wide range of nanoparticles concentrations and exposure times, making it extremely difficult to determine whether the extent of cytotoxicity observed is physiologically significant (Doudi et al., 2013; Rudramurthy et al., 2016). However, biocompatible and non-toxic silver nanoparticles, suitable for biological applications, have been also reported (Gautam and van Veggel, 2013; Rudramurthy et al., 2016). In the case of human intestinal cells, it has been described that silver nanoparticles could be cytotoxic at concentrations of 10 to 50 μg/mL (Chernousova and Epple, 2013; Vazquez-Muñoz et al., 2017). However, at concentrations lower than 10 μg/mL, silver nanoparticles have been reported to be non-toxic to human cells (Chernousova and Epple, 2013). In the present work, the MIC50 value obtained was 19.7 μg/mL, these results indicate that the mean efficacy of GSH-Ag NPs against *Campylobacter* is in a range that is toxic for the three epithelial cell lines studied. This demonstrates the need for further toxicity studies to assess the practical implications of the results obtained and to evaluate other attributes linked to the biological compatibility.

## Conclusions

In conclusion, this study suggests that GSH-Ag NPs could have potential applications to be used as antimicrobial against *Campylobacter*. It has shown to have antimicrobial properties against MDR strains, but very close to or above the toxicity levels determined in this work for human intestinal epithelial cell lines. Although it is clear that further toxicity studies are needed, the emerging practice to combine silver nanoparticles with other compounds is especially promising, because it would make it possible to use lower concentrations of nanoparticles. Particularly in *Campylobacter*, silver nanoparticles could help to enhance antimicrobial strength of antibiotics or other natural bioactive compounds, contributing to reduce therapeutic doses and therefore the putative toxicity. Furthermore, in addition to therapeutic alternatives, these GSH-Ag NPs would be potentially applicable in the different places of the food chain where *Campylobacter* is present, for example in the production and processing of poultry meat, or as an alternative to disinfectants in the *Campylobacter* biofilm control.

## Author contributions

JS: conception, design, acquisition, analysis, and interpretation of data for the work, edition the manuscript and preparation of the tables and figures. IZ-P: analysis and interpretation of data for the work. DG: analysis and interpretation of data for the work. MM-A: conception, design, and interpretation of data for the work. AM-R: conception, design, analysis, interpretation of data for the work, edition the manuscript and preparation the tables and figures.

### Conflict of interest statement

The authors declare that the research was conducted in the absence of any commercial or financial relationships that could be construed as a potential conflict of interest.
